# Cardiovascular effects of bufotenin on human 5-HT_4_ serotonin receptors in cardiac preparations of transgenic mice and in human atrial preparations

**DOI:** 10.1007/s00210-023-02414-8

**Published:** 2023-02-09

**Authors:** Joachim Neumann, Nils Schulz, Charlotte Fehse, Karyna Azatsian, Aneta Čináková, Margaréta Marušáková, Britt Hofmann, Ulrich Gergs

**Affiliations:** 1 Institute for Pharmacology and Toxicology, Medical Faculty, Martin Luther University Halle-Wittenberg, D-06097 Halle, Germany; 2 Faculty of Pharmacy, Gdansk Medical University, Gdansk, Poland; 3 Department of Pharmacology and Toxicology, Faculty of Pharmacy, Comenius University, Bratislava, Slovakia; 4 Cardiac Surgery, Medical Faculty, Martin Luther University Halle-Wittenberg, D-06097 Halle, Germany; 5 Institut für Pharmakologie und Toxikologie, Medizinische Fakultät, Martin-Luther-Universität Halle-Wittenberg, Magdeburger Str. 4, 06112 Halle, Germany

**Keywords:** Serotonin, Bufotenin, Tryptamine, Tropisetron, Pargyline, 5-HT_4_-receptor, Inotropy, Chronotropy, Transgenic mice, Human atrium

## Abstract

It is unclear whether bufotenin (= N,N-dimethyl-serotonin = 5-hydroxy-N,N-dimethyl-tryptamine), a hallucinogenic drug, can act on human cardiac serotonin 5-HT_4_ receptors. Therefore, the aim of the study was to examine the cardiac effects of bufotenin and for comparison tryptamine in transgenic mice that only express the human 5-HT_4_ receptor in cardiomyocytes (5-HT_4_-TG), in their wild-type littermates (WT) and in isolated electrically driven (1 Hz) human atrial preparations. In 5-HT_4_-TG, we found that both bufotenin and tryptamine enhanced the force of contraction in left atrial preparations (pD2 = 6.77 or 5.5, respectively) and the beating rate in spontaneously beating right atrial preparations (pD2 = 7.04 or 5.86, respectively). Bufotenin (1 µM) increased left ventricular force of contraction and beating rate in Langendorff perfused hearts from 5-HT_4_-TG, whereas it was inactive in hearts from WT animals, as was tryptamine. The positive inotropic and chronotropic effects of bufotenin and tryptamine were potentiated by an inhibitor of monoamine oxidases (50 µM pargyline). Furthermore, bufotenin concentration- (0.1–10 µM) and time-dependently elevated force of contraction in isolated electrically stimulated musculi pectinati from the human atrium and these effects were likewise reversed by tropisetron (10 µM). We found that bufotenin (10 µM) increased the phosphorylation state of phospholamban in the isolated perfused hearts, left and right atrial muscle strips of 5-HT_4_-TG but not from WT and in isolated human right atrial preparations. In summary, we showed that bufotenin can increase the force of contraction via stimulation of human 5-HT_4_ receptors transgenic mouse cardiac preparations but notably also in human atrial preparations.

## Introduction

Serotonin (5-hydroxytryptamine, 5-HT) induces a positive inotropic effect and a relaxant effect in the human heart via human 5-HT_4_ receptors (Kaumann and Levy 2006; Neumann et al. 2017). Studies on isolated pig heart preparations have found that 5-HT can increase the force of contraction and frequency via porcine 5-HT_4_ receptors (Kaumann 1990; Villalón et al. 1990). Only in human and pig but not in other mammalian hearts like mouse, cat, rat, dog, or rabbit 5-HT can augment force and beating rate via 5-HT_4_ receptors (Kaumann and Levy 2006; Neumann et al. 2017). In order to have a small animal model of the human 5-HT_4_ receptor, we had established a transgenic mouse, which expresses the human 5-HT_4_ receptor in its cardiomyocytes [5-HT_4_-TG, (Gergs et al. 2010)]. Using a promoter specific for cardiomyocytes, the transgenic human 5-HT_4_ receptor is only expressed in the heart and therein only in cardiomyocytes as shown by immunohistochemistry (Gergs et al. 2010). Exogenously applied serotonin or endogenously in the heart produced serotonin elevates force of contraction in isolated atrial and ventricular cardiac preparations of 5-HT_4_-TG, but not in cardiac preparations of wild-type mice (Gergs et al. 2010, 2013, 2017a, 2017b; Keller et al. 2018; Neumann et al. 2019).

**Fig. 1:**
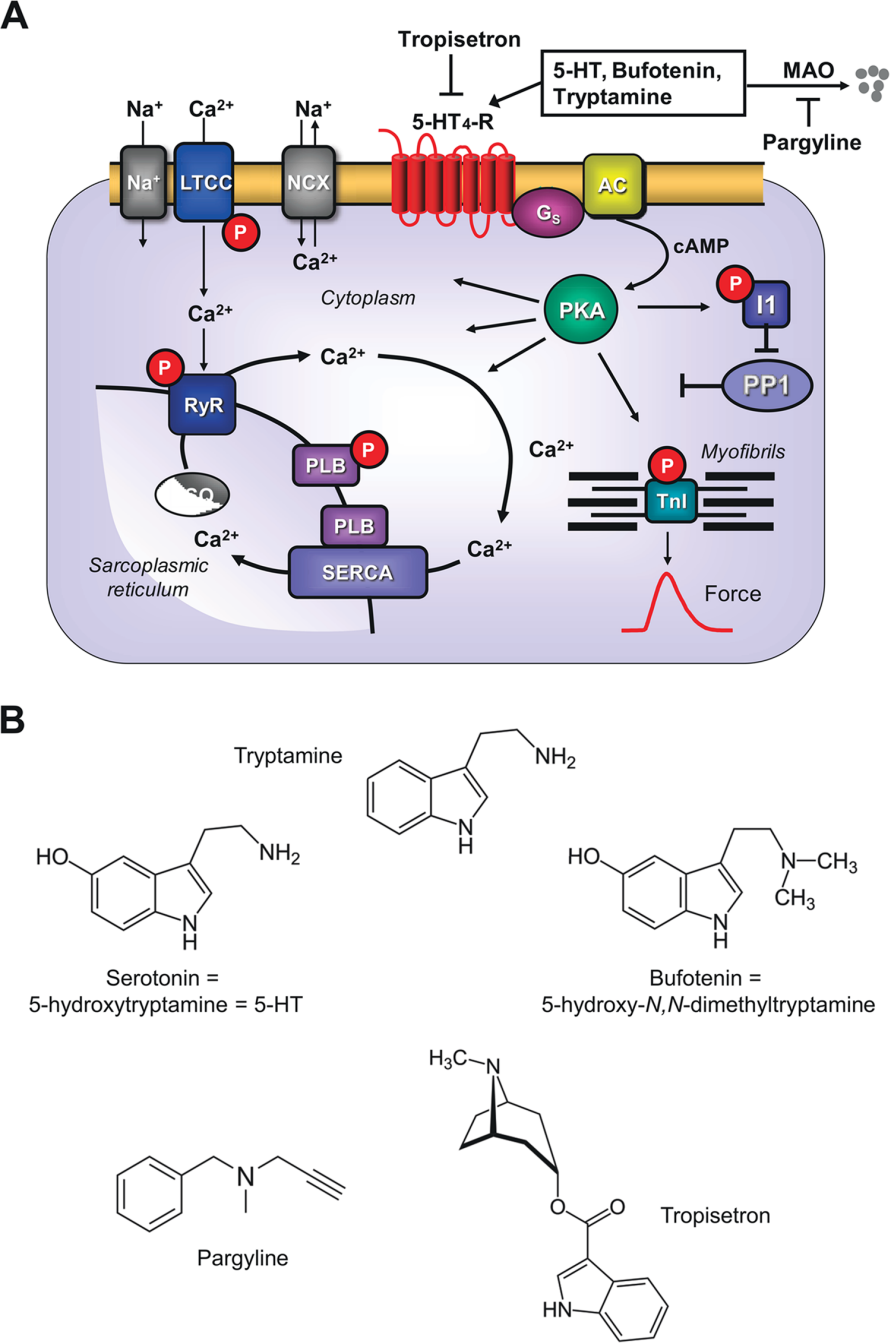
**A** Scheme. Putative mechanism(s) of signal transduction of cardiac 5-HT_4_ receptors. 5-HT_4_ receptors via stimulatory G-proteins (Gs) activate adenylyl cyclases (AC) which enhance the 3′-5′cyclic adenosine-phosphate (cAMP) levels in compartments of the cardiomyocyte and activate cAMP-dependent protein kinases (PKA) which increase the phosphorylation state and thereby the activity of various regulatory proteins in the cell. PKA-stimulated phosphorylation increases the current through the L-type Ca^2+^ channel (LTCC) and/or release of Ca^2+^ from the sarcoplasmic reticulum (SR) via the cardiac ryanodine receptor (RYR2); both processes would increase force of contraction by increasing the Ca^2+^ acting on myofilaments. In diastole, Ca^2+^ is pumped via the SR-Ca^2+^ ATPase (SERCA) from the cytosol into the SR. Activity of SERCA is increased by the phosphorylation of phospholamban (PLB). The latter effect might also follow from inhibition of PP1 (a serine/threonine phosphatase: PP) activity by increased phosphorylation state and thus activation of I-1 (a specific inhibitory protein of PP1) which will lead to decreased activity of PP1. Reduced activity of PP1 increases the phosphorylation of additional proteins and thus increases the Ca^2+^ sensitivity of myofilaments by dephosphorylation of the myosin light chains in the myofilaments which increases force of contraction. Thus, 5-HT_4_ receptors increase the Ca^2+^ sensitivity of myofilaments. **B** Structures of drugs used in this study

Now, bufotenin (5-hydroxy-dimethyltryptamine) is structurally related to serotonin: bufotenin can be regarded as a dimethylated serotonin (Fig. 1). Hence, it is not surprising that based on this similarity, bufotenin can bind to serotonin receptors. Indeed, bufotenin and tryptamine bind both to 5-HT_2A_ and 5-HT_2C_ receptors, with bufotenin being more potent than tryptamine (Almaula et al. 1996). Agonist binding to 5-HT_2A_ receptors is thought to explain the hallucinogenic effects of bufotenin and tryptamine (Titeler et al. 1988). Moreover, bufotenin binds potently to 5-HT_1A_, 5-HT_1B_, and 5-HT_1D_ receptors and tryptamine is a known 5-HT_4_ receptor agonist (Dumuis et al. 1988). Others had shown that bufotenin and tryptamine (interestingly only in the presence of 50 µM pargyline, an inhibitor of monoamine oxidase (MAO) activities) exerted positive chronotropic effects in isolated spontaneously beating right atrial preparations from pigs, mediated by porcine 5-HT_4_ receptors (Medhurst and Kaumann 1993). However, as far as we are aware, inotropic effects of bufotenin in isolated mammalian heart preparations (isolated atrium or isolated heart), particularly human heart preparations, have not been reported.

Bufotenin was first isolated to purity in the city of Prague from toad skin (in Latin *bufo* means toad) in 1920 by Hans Handovsky (Handovsky 1920), the correct structural formula (they called it “5-Oxy-indolyl-äthyl-dimethylamin”) was found in Munich by Heinrich Wieland (Chilton et al. 1979; Wieland et al. 1934). Handovsky (1920) and Wieland et al. (1934) used extracts of parotids from 1000 toads or 10,000 toads (*Bufo vulgaris*), respectively. The chemical synthesis of bufotenin was reported in 1935 (Hoshino and Shimodaira 1935).

The use of hallucinogenic compounds like bufotenin was classically described by the German pharmacologist Louis Lewin (Lewin 1924). The older literature on toxins in toad extracts was collected by Faust (Faust 1902): fatal intoxications of adult men by toads as early as 1575 were reported by Ambroise Paré (see (Faust 1902)). Faust called the toxin “bufonin” which we now regard as “bufotenin” or “bufotenine” (Faust 1902).

Bufotenin not only occurs in animals like toads but also presents itself in plants: Shamans in French Guiana use latex from *Brosimum* *acutifolium* to obtain hallucinogenic mixtures that were found to contain bufotenin (Moretti et al. 2006). Another botanical source for bufotenin is the seeds of *Anandenanthera peregrina* that is found in northern parts of South America namely Columbia, Venezuela, Ecuador, Peru, and Brazil where it is used in the galenic forms of snuffs, fumatories, enemas, masticatories, and potions for ritual purposes (review (Ott 2001)).

Puzzlingly, bufotenin was not only found in toads but also in the human body (Forsström et al. 2001). It might be formed enzymatically by a methyltransferase from serotonin (Fig. 1) in human neuronal cells (Kärkkäinen et al. 2005).

Bufotenin has been suggested to underlie the tale of the Frog Prince as published as a fairy tale by the Grimm brothers (Siegel and McDaniel 1991): in fairy tales, licking the skin of a frog or kissing frogs can release bufotenin and that leads to hallucinations and imagining a prince (Siegel and McDaniel 1991).

5-Hydroxytryptamine (5-HT) and 5-hydroxy-dimethyltryptamine (bufotenin) contain a tryptamine moiety (Fig. 1). Hence, it seemed reasonable based on the related chemical structure to study tryptamine in comparison with bufotenin in some experiments. At least in rat liver mitochondria, tryptamine is metabolized by both MAO A and MAO B (Suzuki et al. 1981) and others reported that tryptamine was inotropically inactive if no MAO inhibitor (in their case pargyline) was added in organ bath experiments (Medhurst and Kaumann 1993).

The aim of the present work was to gain further insight into the cardiac effects of two putative 5-HT_4_ receptor agonists: bufotenin and tryptamine hitherto only studied for their chronotropic effects in isolated porcine right atrium (Medhurst and Kaumann 1993).

Thus, in this study, we tested the hypothesis that bufotenin and tryptamine act as agonists and/or antagonists on human cardiac 5-HT_4_ receptors.

## Materials and methods

### Transgenic mice

A mouse with cardiomyocyte-specific expression of the human 5-HT_4(a)_ receptor has been generated in our laboratory (Gergs et al. 2010). The cardiac myocyte-specific expression was achieved by the use of the α-myosin heavy chain promoter. The age of the animals studied in the atrial contraction experiments was around 4 months. Both genders were studied. All mice were housed under conditions of optimum light, temperature, and humidity with food and water provided ad libitum. The investigation conformed to the *Guide for the Care and Use of Laboratory Animals* as published by the National Research Council (2011). The animals were handled and maintained according to the approved protocols of the Animal Welfare Committee of the University of Halle-Wittenberg, Halle, Germany.

### Contractile studies in mice

In brief, the right or left atrial preparations from the mice were isolated and mounted in organ baths as previously described (Gergs et al. 2013; Neumann et al. 2003). The bathing solution of the organ baths contained 119.8 mM NaCI, 5.4 mM KCI, 1.8 mM CaCl_2_, 1.05 mM MgCl_2_, 0.42 mM NaH_2_PO_4_, 22.6 mM NaHCO_3_, 0.05 mM Na_2_EDTA, 0.28 mM ascorbic acid, and 5.05 mM glucose. The solution was continuously gassed with 95% O_2_ and 5% CO_2_ and maintained at 37 °C and pH 7.4 (Kirchhefer et al. 2004; Neumann et al. 1998, 2003). Spontaneously beating right atrial preparations from mice were used to study any chronotropic effects.

The drug application was as follows. After equilibration was reached, 1 nM to 10 µM 5-HT, bufotenin or tryptamine were added to organ baths of 10 ml volume that housed the left atrium or right atrium, with the intent to establish concentration response curves.

### Contractile studies on human preparations

The contractile studies on human right atrial preparations were done using the same setup and buffer as used in the mouse studies (see “Contractile studies in mice” section). The samples were obtained from male patients that underwent a bypass surgery (Table 1). Drug therapy included β-receptor blockers, calcium channel blockers, angiotensin-converting enzyme inhibitors/angiotensin receptor blockers, diuretics, direct oral anti-coagulants, metformin, statins, and acetyl salicylic acid. Our methods used for atrial contraction studies in human samples have been previously published and were not altered in this study (Boknik et al. 2019; Gergs et al. 2009, 2017b, 2018). This study complies with the Declaration of Helsinki and has been approved by the local ethics committee (hm-bü 04.08.2005) and all patients gave informed consent.

**Table 1:** Clinical data of the patients (all male) included in the study

Patient ID	Age (years)	NYHA class	CCS angina grading scale	LVEF (%)	Cardiac catheterization findings
#1	72	III-IV	III	40	3 vessel CHD, aortic valve stenosis
#2	52	III	III	60	3 vessel CHD
#3	74	III	III	41	3 vessel CHD
#4	61	III	III	55	3 vessel CHD
#5	64	III	III	60	3 vessel CHD
Mean ± SD	64.6 ± 8.9			51.2 ± 10.0	

*NYHA* New York Heart Association, *CCS* Canadian Cardiovascular Society, *LVEF* left ventricular ejection fraction, *CHD* coronary heart disease

### Western blotting

The homogenization of the samples, protein measurements, SDS polyacrylamide gel electrophoresis, primary and secondary antibody incubation and quantification were performed following our previously established protocols (Boknik et al. 2018; Gergs et al. 2009, 2019a, 2019c).

### Langendorff hearts

We followed previously reported methods for the treatment of the animals. This included the removal of the heart and lungs from the thoracic cavity, placing the dissected heart onto a cannula, the use of custom-made equipment, the buffer composition, quantification of the force from the left ventricular apex, digitization of the recordings, and freeze clamping the hearts (Boknik et al. 2019; Gergs et al. 2019b).

### Data analysis

Data shown are mean ± standard error of the mean. Statistical significance was estimated using the analysis of variance (ANOVA) followed by Bonferroni’s post-test or a Student’s *t*-test as appropriate. A *p*-value  <0.05 was considered to be significant.

### Drugs and materials

The drugs isoprenaline-bitartrate salt, serotonin (5-HT) hydrochloride, bufotenin, pargyline and tryptamine were purchased from Sigma-Aldrich (Merck, Darmstadt, Germany). All other chemicals were of the highest purity grade commercially available. Deionized water was used throughout the experiments. Stock solutions were prepared fresh daily.

## Results

### Studies in the isolated atria from mice

**Fig. 2:**
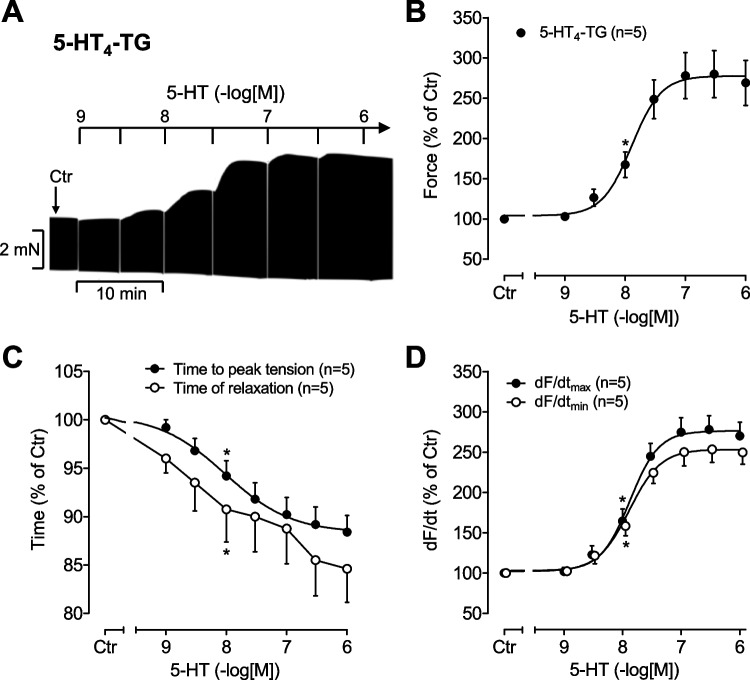
Serotonin (5-HT) increases contractility in atrial preparations of mice overexpressing 5-HT_4_ receptors. **A** Representative original recording of a concentration response curve for 5-HT in an isolated electrically stimulated (1 Hz) left atrial preparation from 5-HT_4_-TG. **B** Concentration- and time-dependent positive inotropic effect of 5-HT in isolated electrically stimulated (1 Hz) atrial preparations from 5-HT_4_-TG. Basal force of contraction amounted to 2.74 ± 0.29 mN. **C** Concentration effect of 5-HT on time to peak tension and on time of relaxation in isolated electrically stimulated (1 Hz) left atrial preparations from 5-HT_4_-TG. Basal time to peak tension amounted to 14.18 ± 0.3 ms and basal time of relaxation amounted to 31.82 ± 1.69 ms. **D** Concentration-dependent effect of 5-HT on maximum rate of tension development (dF/dt max) and minimum rate of tension development (dF/dt min) in isolated electrically stimulated (1 Hz) left atrial preparations from 5-HT_4_-TG. Basal maximum rate of tension development amounted to 177.06 ± 20.74 mN/s and basal minimum rate of tension development amounted to − 99.6 ± 11.31 mN/s. *Indicates the first significant difference versus control (Ctr = before drugs addition). Numbers in brackets = number of experiments

5-HT increased the force of contraction in the left atrium from 5-HT_4_-TG (Fig. 2). This was reported before (Gergs et al. 2010, 2013) but here, new experiments were performed on the same experimental days as the studies on bufotenin. This is seen in an original recording (Fig. 2A) and summarized in Fig. 2B. At the same time, 5-HT concentration-dependently shortened time to peak tension and time of relaxation (Fig. 2C). Likewise, 5-HT concentration-dependently augmented the maximum rate of tension development (dF/dt_max_) and the minimum rate of tension development (dF/dt_min_) (Fig. 2D). In isolated right atrial preparations, 5-HT concentration-dependently elevated the beating rate in 5-HT_4_-TG (data not shown).

**Fig. 3:**
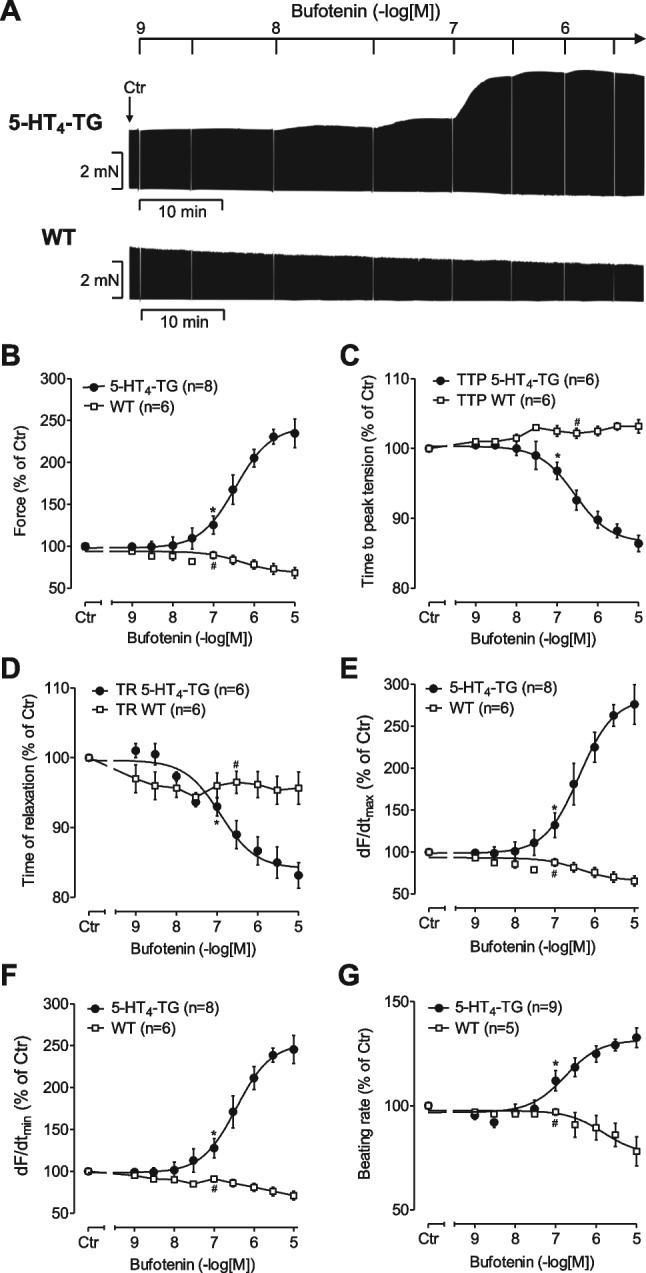
Bufotenin increases contractility in atrial preparations of mice overexpressing 5-HT_4_ receptors. **A** Representative original recordings of a concentration response curve for bufotenin in an isolated electrically stimulated (1 Hz) left atrial preparation from 5-HT_4_-TG and for comparison from WT. **B** Concentration-dependent positive inotropic effect of bufotenin in isolated electrically stimulated (1 Hz) atrial preparations from 5-HT_4_-TG (circles). There were no effects of bufotenine in WT preparations (squares). Basal force of contraction amounted to 2.82 ± 0.42 mN. **C** Concentration-dependent effect of bufotenin on time to peak tension (TTP) and **D** on time of relaxation (TR) in isolated electrically stimulated (1 Hz) atrial preparations from 5-HT_4_-TG (circles) and WT (squares). Basal TTP amounted to 14.86 ± 0.31 ms and basal TR amounted to 31.18 ± 1.77 ms. **E** Concentration-dependent effect of bufotenin on maximum and **F** minimum rate of tension development (dF/dt max and min) in isolated electrically stimulated (1 Hz) atrial preparations from 5-HT_4_-TG (circles) and WT (squares). Basal maximum rate of tension development amounted to 174.3 ± 31.87 mN/s and basal minimum rate of tension development amounted to − 99.2 ± 16.31 mN/s. **G** Concentration-dependent chronotropic effect of bufotenin in isolated spontaneously beating atrial preparations from 5-HT_4_-TG (circles) and WT (squares). Basal rate of contraction amounted to 310 ± 22 beats per minute. *Indicates the first significant difference versus control (Ctr = before drugs addition), #indicates the first significant difference versus 5-HT_4_-TG. Numbers in brackets = number of experiments

As a new step, we wanted to determine whether bufotenin also exerted positive inotropic effects in 5-HT_4_-TG. We found that bufotenin (original recordings: Fig. 3A) raised force of contraction in a concentration- and time-dependent manner in 5-HT_4_-TG but not in WT; these results are summarized in Fig. 3B. It is apparent that bufotenin increased force of contraction and the beating rate slower than 5-HT (compare Figs. 2A and 3A). In addition, in isolated electrically driven left atrial preparations from 5-HT_4_-TG, bufotenin shortened the time to peak tension and the time of relaxation compared to tissue obtained from WT animals (Fig. 3C). The maximum positive first derivate of the developed force and the minimum first derivate (Fig. 3D) showed a similar pattern as the force (Fig. 3B). However, the increases due to the bufotenin on the maximum positive first derivate of the developed force were more pronounced than the increases in force of contraction (Fig. 3D versus B). Finally, bufotenin concentration- and time-dependently increased the beating rate of the right atrium from 5-HT_4_-TG and not WT (Fig. 3E). The potency was assessed using EC_50_-values and are plotted in Table 2. The effects of bufotenin in left and right atrium of 5-HT_4_-TG were shifted to the left by 50 µM pargyline, a MAO inhibitor.

**Table 2:** Summary of the negative decadic logarithms of EC_50_ values (= pD2 values) in atrial preparations from 5-HT_4_-TG. Data are separated according to the tissue tested: either left or right atrium of 5-HT_4_-TG. Number of animals ranged from 4 to 7

Tissue	Parameter	5-HT	Bufotenin	Tryptamine	Tryptamine + pargyline
LA	Force of contraction	7.94 ± 0.32 *N* = 4	6.77 ± 0.52 * *N* = 5	4.57 ± 0.92 *^#^ *N* = 5	5.48 ± 0.64 *^#^ *N* = 5
LA	TTP	8.07 ± 0.24 *N* = 5	6.45 ± 0.47 * *N* = 5	5.18 ± 0.53 *^#^ *N* = 5	6.12 ± 0.38 *^+^ *N* = 5
LA	TR	8.62 ± 0.83 *N* = 4	6.31 ± 1.65 * *N* = 5	5.54 ± 0.87 * *N* = 5	6.45 ± 0.99 *N* = 5
LA	dF/dt_max_	7.87 ± 0.25 *N* = 4	6.9 ± 0.73 * *N* = 5	4.73 ± 0.89 *^#^ *N* = 7	5.47 ± 0.59 *^#^ *N* = 5
LA	dF/dt_min_	7.91 ± 0.34 *N* = 4	6.92 ± 0.7 * *N* = 5	4.73 ± 0.79 *^#^ *N* = 7	5.51 ± 0.45 *^#+^ *N* = 5
RA	Beating rate	7.05 ± 5.02 *N* = 4	7.04 ± 1.08 *N* = 6	5.27 ± 0.53 *^#^ *N* = 5	5.98 ± 1.63 * *N* = 5

*LA* left atrium, *RA* right atrium, *TTP* time to peak, *TR* time of relaxation, *dF/dt* maximum and minimum of the first derivative of force of contraction. ANOVA: *p < 0.05 vs. 5-HT; ^#^*p* < 0.05 vs. bufotenin; ^+^*p* < 0.05 vs. tryptamine

**Fig. 4:**
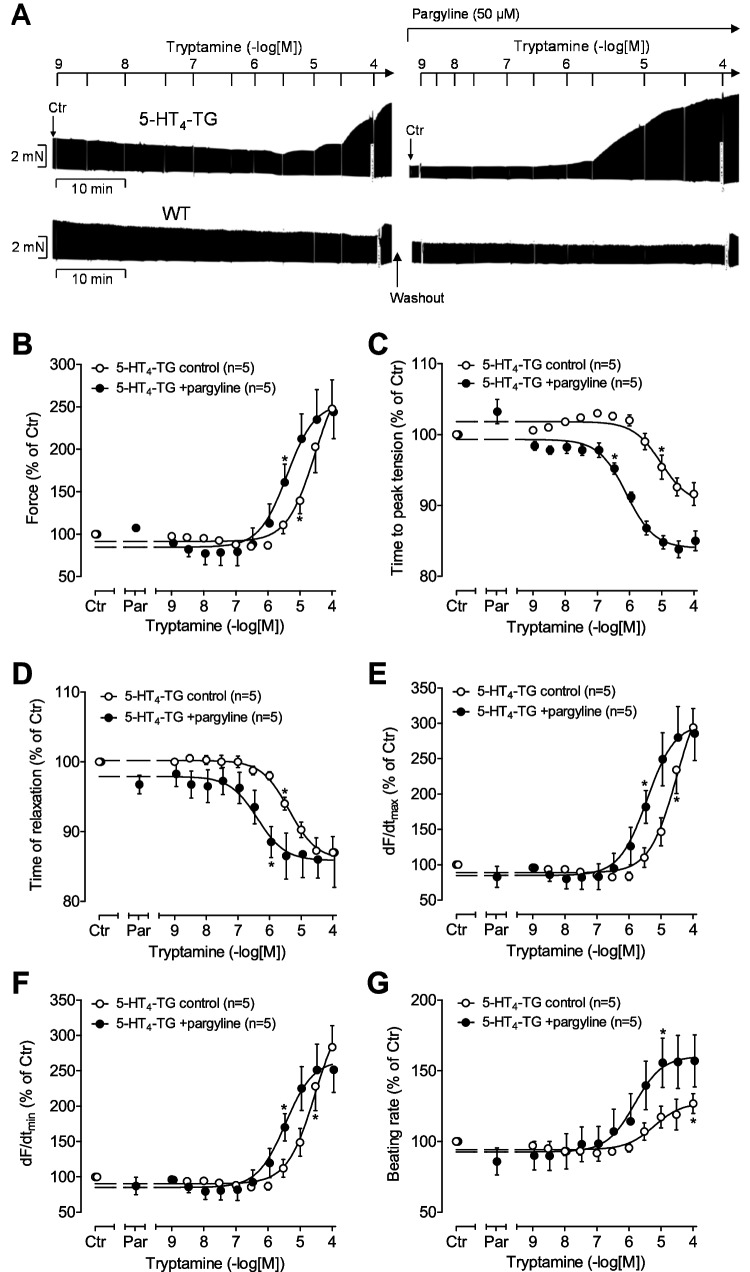
Tryptamine increases contractility in atrial preparations of mice overexpressing 5-HT_4_ receptors. **A** Representative original recordings of a concentration response curve for tryptamine in isolated electrically stimulated (1 Hz) left atrial preparations from 5-HT_4_-TG and for comparison from WT. **B** Concentration- and time-dependent positive inotropic effect of tryptamine alone (open circles) or in the presence of 50 µM pargyline (filled circles) in isolated electrically stimulated (1 Hz) atrial preparations from 5-HT_4_-TG. Basal force of contraction amounted to 2.72 ± 0.88 mN. **C** Concentration-dependent effect on time to peak tension of tryptamine alone (open circles) or in the presence of 50 µM pargyline (filled circles). Basal time to peak tension amounted to 14.51 ± 0.29 ms. **D** Concentration-dependent effect on time of relaxation of tryptamine alone (open circles) or in the presence of 50 µM pargyline (filled circles). Basal time of relaxation amounted to 37.91 ± 8.46 ms. **E** Concentration-dependent effect on maximum rate of tension development (dF/dt max) of tryptamine alone (open circles) or in the presence of 50 µM pargyline (filled circles). Basal maximum rate of tension development amounted to 171.84 ± 49.42 mN/s. **F** Concentration-dependent effect on minimum rate of tension development (dF/dt min) of tryptamine alone (open circles) or in the presence of 50 µM pargyline (filled circles). Basal minimum rate of tension development amounted to  −91.71 ± 20.25 mN/s. **G** Concentration-dependent chronotropic effect in isolated spontaneously beating right atrial preparations of tryptamine alone (open circles) or in the presence of 50 µM pargyline (filled circles). Basal beating rate amounted to 325 ± 29 beats per minute. *Indicates the first significant difference versus control (Ctr = before drugs addition). Numbers in brackets = number of experiments

As seen in Fig. 4A (original recording), tryptamine like 5-HT or bufotenin increased force of contraction in left atrium from 5-HT_4_-TG but not from WT. In contrast to work of others (Medhurst and Kaumann 1993), in 5-HT_4_ mouse atrium, tryptamine in the absence of any monoamine deaminase (MAO) inhibitor was able to increase the force of contraction and the beating rate, but addition of pargyline (50 µM, the same drug and concentration as used by (Medhurst and Kaumann 1993)) as a MAO inhibitor, potentiated the effects of tryptamine (Table 2). This is plotted in Fig. 4B (WT graphs have been omitted for better clarity). Tryptamine alone and in the additional presence of pargyline, shortened the time of tension development (Fig. 4C), and the time of tension relaxation (Fig. 4D) in left atrium from 5-HT_4_-TG but not from WT. Tryptamine alone and in the additional presence of pargyline increased the maximum positive first derivate of the developed force dF/dt_max_ (Fig. 4E) and minimum first derivate of the developed force dF/dt_min_ (Fig. 4F). Tryptamine alone and in the additional presence of pargyline increased the beating rate in right atrium from 5-HT_4_-TG but not from WT (Fig. 4G). Of note, tryptamine was always more potent in the presence compared to the absence of pargyline. The EC_50_ of 5-HT, bufotenin, tryptamine in the absence and presence of pargyline are plotted in Table 2 to facilitate comparison.

**Fig. 5:**
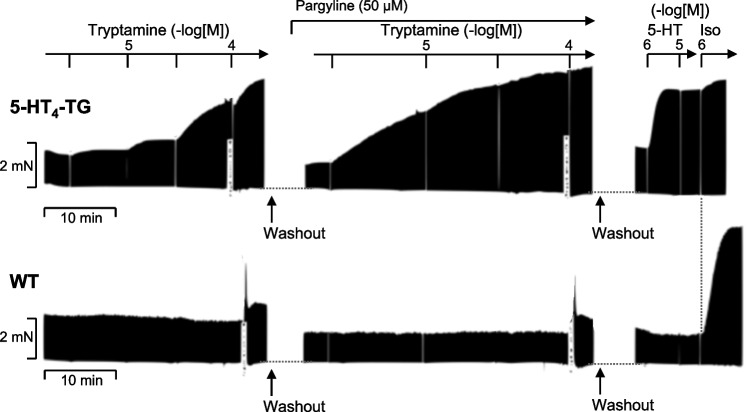
Efficacy of tryptamine. Original recordings: Concentration- and time-dependent positive inotropic effect of tryptamine (plus 50 µM pargyline) in isolated electrically stimulated (1 Hz) left atrial preparations from 5-HT_4_-TG. After completion, 1 µM and 10 µM 5-HT were added and thereafter 1 µM isoproterenol (Iso). Ordinate depicts measured force in milli Newton (mN). Horizonal bar indicates time in minutes (min). Data are tabulated and statistically evaluated in Table 3

We also wanted to determine whether tryptamine acts as full agonist on 5-HT_4_ receptors. Therefore, at the end of the concentration response curves of tryptamine, 5-HT (1 and 10 µM) was given. As seen in the original tracings in Fig. 5 and summarized in Table 2, tryptamine was nearly a full agonist compared to 5-HT, but was less potent. Both 5-HT and tryptamine were less effective than isoproterenol to increase force of contraction and beating rate (Fig. 5).

**Table 3:** Efficacy of bufotenin and tryptamine in comparison with 1 µM of serotonin (5-HT) are evaluated. One micrometer 5-HT is maximally effective to increase force of contraction or beating rate in LA or RA of 5-HT_4_-TG, respectively. In these experiments, first a concentration response curve to bufotenin/tryptamine was cumulatively established then additionally 5-HT was applied to the isolated atrium. In this way, it was possible to compare the maximum stimulation of 5-HT_4_-TG receptors. Number of animals ranged from 5 to 7

Tissue	Parameter	5-HT (1 µM)	Bufotenin (3 µM)	Tryptamine (100 µM)	Tryptamine (100 µM) + pargyline (50 µM)
LA	Force (mN) and in % of 1 µM 5-HT	7.07 ± 0.43, *n* = 5	6.45 ± 0.75, *n* = 6, 91% ± 5%	5.74 ± 0.84, *n* = 5, 81% ± 7%	4.88 ± 0.71, *n* = 5, 69% ± 5%
LA	TTP (ms) and in % of 1 µM 5-HT	12.53 ± 0.11, *n* = 5	12.84 ± 0.15, *n* = 5, 98% ± 0%	13.28 ± 0.07, *n* = 5, 94% ± 1%	13.04 ± 0.04, *n* = 5, 96% ± 1%
LA	TR (ms) and in % of 1 µM 5-HT	26.77 ± 0.71, *n* = 5	26.08 ± 1.23, *n* = 5, 103% ± 2%	27.61 ± 1.25, *n* = 5, 97% ± 2%	26.13 ± 1.27, *n* = 5, 102% ± 2%
LA	dF/dt_max_ (mN/s) and in % of 1 µM 5-HT	507.42 ± 32.86, *n* = 5	440.7 ± 61.25, *n* = 5, 87% ± 6%	393.56 ± 52.26, *n* = 5, 78% ± 5%	339.02 ± 43.4, *n* = 5, 67% ± 4%
LA	dF/dt_min_ (mN/s) and in % of 1 µM 5-HT	− 264.14 ± 16.93, *n* = 5	− 233.6 ± 29.54, *n* = 5, 88% ± 6%	− 217.68 ± 24.09, *n* = 5, 82% ± 4%	− 186.48 ± 20, *n* = 5, 71% ± 2%
RA	Beating rate (bpm) and in % of 1 µM 5-HT	369 ± 60, *n* = 5	396 ± 35, *n* = 7, 107% ± 7%	388 ± 41, *n* = 7, 105% ± 5%	336 ± 62, *n* = 7, 91% ± 2%

*LA* left atrium, *RA* right atrium, *TTP* time to peak, *TR* time of relaxation, *dF/dt* maximum and minimum of the first derivative of force of contraction

### Langendorff hearts

Isolated hearts were prepared according to Langendorff (1895). In this study, we found that 1 µM bufotenin increased the force of contraction in isolated Langendorff hearts of 5-HT_4_-TG from 9.74 ± 1.51 to 15.6 ± 1.42 mN, the rate of force development from 315 ± 57 to 653 ± 86 mN/s and the rate of relaxation from 227 ± 43 to 515 ± 43 mN/s (*n* = 5; *p* < 0.05). However, bufotenin was inactive in Langendorff hearts from WT mice (data not shown, *n* = 3).

### Protein phosphorylation

**Fig. 6:**
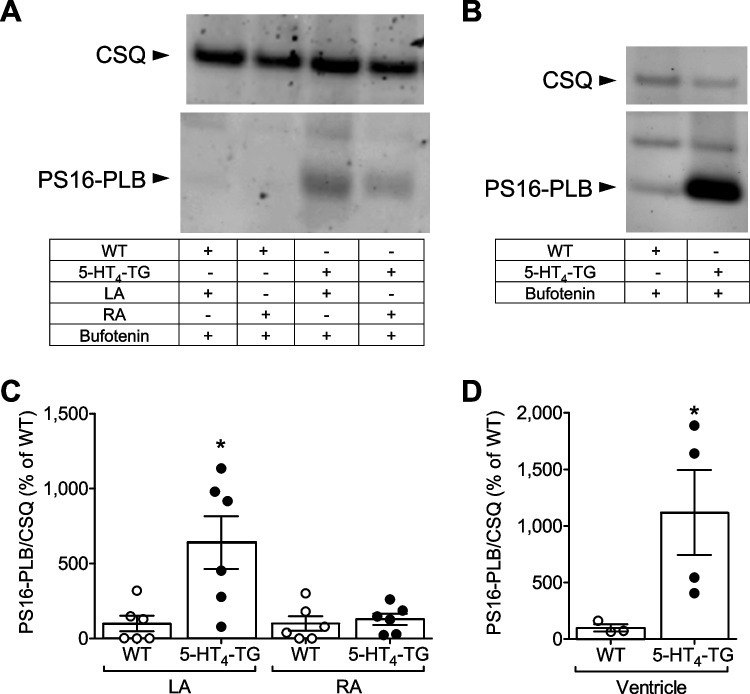
Bufotenin increases phosphorylation of phospholamban (PLB) in the heart. Effect of 1 µM bufotenin on serine 16 phosphorylation of phospholamban (PS16-PLB) in (**A**, **C**) isolated electrically stimulated left atrium (LA) or spontaneously beating right atrium (RA) and in (**B**, **D**) isolated perfused hearts from wild type (WT) and 5-HT_4_-transgenic (5-HT_4_-TG) mice. Typical Western blots are seen on upper parts (**A** and **B**) and bar diagrams below (**C** and **D**) summarize the data. Numbers in columns indicate numbers of experiments. *Indicate significant differences versus WT. As a loading control, we assessed the protein expression of calsequestrin (CSQ) by cutting the lanes of the blot and incubating the lower and upper halves with different primary antibodies

Next, we wanted to understand the putative underlying mechanisms of the contractile effects of bufotenin (Fig. 1). In previous studies, we have shown that 5-HT could increase the phosphorylation state of phospholamban in human and 5-HT_4_-TG preparations but not in WT (Gergs et al. 2009, 2010, 2013). In this study, we used a comparable approach and noted that the force of contraction, rate of relaxation and the phosphorylation state of phospholamban showed a consistent pattern. An increase in the phosphorylation state of phospholamban was noted in freeze-clamped 5-HT_4_-TG left atrial preparations that were treated with bufotenin (Fig. 6). This can be seen in original Western blots from atrial experiments (Fig. 6A) and these data are summarized in Fig. 6C: 1 µM bufotenin increased the phosphorylation state of phospholamban on the amino acid serine 16 in the atria of 5-HT_4_-TG but not of WT. The lower effect noted in 5-HT_4_-TG right atrial preparations (Fig. 6A, C) possibly due to the differences in the beating rate of the preparations (RA: spontaneously beating at 300–400 bpm, LA: paced at 60 bpm). We speculate that this may explain the differences in the phosphorylation state of phospholamban. In order to study the effects of bufotenin in the ventricle, we studied freeze-clamped isolated spontaneously beating retrogradely perfused mouse hearts (Langendorff preparation): 1 µM bufotenin increased the phosphorylation state of phospholamban on serine 16 in isolated hearts from 5-HT_4_-TG but not WT (Fig. 6B and D).

### Human atrial preparations

**Fig. 7:**
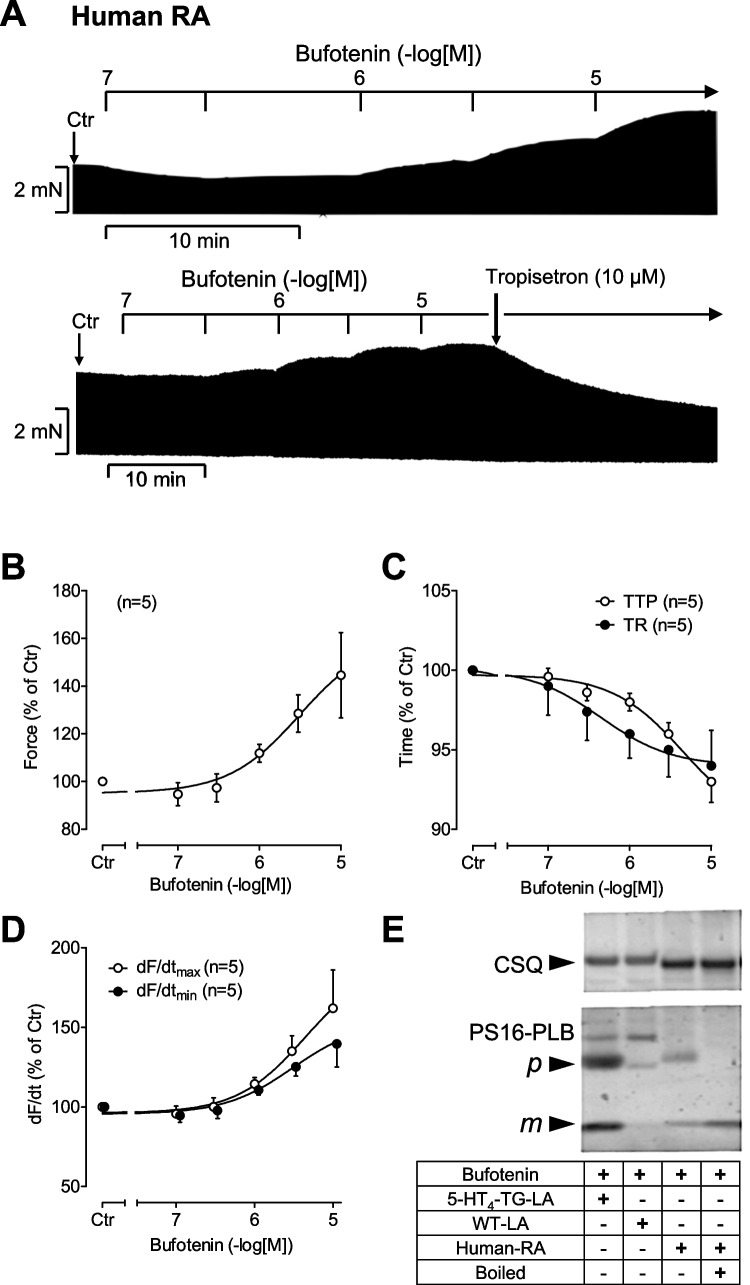
Bufotenin increases force of contraction in the human right atrium (RA). **A** Representative original recordings of a concentration response curve for bufotenin in isolated electrically stimulated (1 Hz) human right atrial preparations. **B** Concentration- and time-dependent positive inotropic effect of bufotenin in isolated electrically stimulated (1 Hz) atrial preparations from human. Basal force of contraction amounted to 3.79 ± 1.6 mN. **C** Concentration-dependent effect of bufotenin on time to peak tension (TTP) and time of relaxation (TR) in isolated electrically stimulated (1 Hz) atrial preparations from human. Basal time to peak tension amounted to 42.89 ± 1.59 ms and basal time of relaxation amounted to 89.22 ± 6.34 ms. **D** Concentration-dependent effect of bufotenin on maximum and minimum rate of tension development (dF/dt max and min) in isolated electrically stimulated (1 Hz) atrial preparations from human. Basal maximum rate of tension development amounted to 80.35 ± 32.21 mN/s and basal minimum rate of tension development amounted to  −49.68 ± 17.94 mN/s. *Indicates the first significant difference versus control (Ctr = before drugs addition), Numbers in brackets = number of experiments, N = number of patients, n = number of trabecules (*N* = 1, *n* = 4). **E** By SDS polyacrylamide gel electrophoresis and Western blotting, the effects of bufotenin on serine 16 phosphorylation of phospholamban (PS16-PLB) were demonstrated. Calsequestrin (CSQ) was monitored as a loading control in these gels. One sample of human cardiac tissue was boiled immediately prior to electrophoresis and shows a mobility shift from the pentameric (*p*) form to the monomeric (*m*) form of PLB and thus proves its identity. The mouse samples are shown for comparison

In isolated paced human atrial trabeculae, bufotenin led to concentration- and time-dependent positive inotropic effects (Fig. 7). This is seen in the original tracings (Fig. 7A). Moreover, it is apparent in the lower tracing that Tropisetron, additionally applied, revoked the positive inotropic effect of bufotenin (Fig. 7A). Data for force of contraction are summarized in Fig. 7B. The positive inotropic effects of bufotenin were accompanied by a decrease of the time parameters of the contraction (Fig. 7C) and an elevation in the rate of force development and an increase in the rate of relaxation (Fig. 7D). Furthermore, in electrically stimulated human right atrial preparations, bufotenin increased the phosphorylation state of phospholamban (Fig. 7E, as seen in an original Western blot). Calsequestrin was monitored as a loading control in these gels. One sample of human cardiac tissue was boiled immediately prior to electrophoresis and shows a mobility shift consistent with the known physiochemical behavior of phospholamban and thus proves its identity. The mouse samples are shown for comparison.

## Discussion

Whereas it was shown before that tryptamine derivatives including bufotenin can mediate tachycardia via 5-HT_4_ receptors in piglet isolated right atrium (Medhurst and Kaumann 1993), the main new findings in the present study are that bufotenin can also increase cardiac force of contraction if 5-HT_4_ receptors are functionally present, which was the case in transgenic mice and human atrium. These functional effects are accompanied and probably, at least in part, mediated by phosphorylation of phospholamban.

As a model system, we used cardiac preparations from mice overexpression human 5-HT_4_ receptors. We generated this model several years ago and used it repeatedly to study putative cardiac effects on 5-HT_4_ receptors. The exact receptor density of 5-HT_4_ receptors is at present speculative. Using radioligands or Western blots, we did not detect the protein for the 5-HT_4_ receptors in a convincing way. Nevertheless, this model was useful to show, for instance, that prokinetic drugs like metoclopramide, prucalopride, and cisapride can stimulate cardiac 5-HT_4_ receptors (Keller et al. 2018; Neumann et al. 2021a). The model was also useful to investigate homologous and heterologous functional desensitization of the human 5-HT_4_ receptors in atrium and ventricle (Gergs et al. 2017a). In this model, we have also studied which phosphodiesterases are functional antagonistic of 5-HT in the heart (Neumann et al. 2019). We also used this model to study proarrhythmic effects of 5-HT under normothermia and hypothermia via 5-HT_4_ receptors (Gergs et al. 2021b; Keller et al. 2018). Conversely, we used the predictive possibilities of this mouse model to falsify the hypothesis that domperidone, another prokinetic drug, can stimulate cardiac 5-HT_4_ receptors in human atrium (Neumann et al. 2021a). We have likewise utilized this model to study in some detail the effect of hypertrophy, sepsis, hypoxia, and ischemia on the cardiac function of 5-HT_4_ receptors (Gergs et al. 2021a). Likewise, we have used this model to predict an interaction of 5-HT_4_ and H_2_ histamine that we then could confirm in human atrial preparations (Neumann et al. 2021b). We have shown early on that stimulation of 5-HT_4_ receptors occurs on cardiomyocytes in transgenic mice and leads to increase in currents through L-type Ca^2+^ channels and increased levels of free calcium ions in the cytosol in systole (Gergs et al. 2010). The receptor is active in vivo as injection of 5-HT increased ventricular function in living animals assessed by echocardiography (Gergs et al. 2010) and increased the beating rate in freely moving transgenic mice as measured by telemetry (Gergs et al. 2013). We have also used this model to present evidence for the formation and degradation of 5-HT in the mammalian heart (Gergs et al. 2017b). Hence, the receptor is operational and the model is useful to predict effects in the human heart: this was the reason why we used this mouse model in the present paper.

In the very first paper with pure bufotenin, bufotenin was studied for its cardiac effects: while bufotenin (even at high doses) did not alter force of contraction in the isolated frog heart, bufotenin reduced heart rate in the frog (Handovsky 1920). Intravenous injections of bufotenin in dogs, cats, or rabbits (that we now know not to express 5-HT_4_ receptors in the heart) increased blood pressure (due to constriction of arteries) but shortly after the injection the animals died (Handovsky 1920). The name bufotenin was coined by French researchers, who investigated toad extracts (bufoténine (Phisalix and Bertrand 1893)) and noted that they increase blood pressure. Knowledge of toad toxins predates modern research. As we now know, bufotenin containing extracts were used in, for instance, Bolivia around AD 1000 in combination with its natural occurring isomer called psilocin for ritual purposes (Miller et al. 2019).

The present study showed that bufotenin and tryptamine exerted a concentration-dependent positive inotropic effect, were less potent than 5-HT on human 5-HT_4_ receptors. Thus, we present data that bufotenin is a full agonist and tryptamine a partial agonist, similar to cisapride, on 5-HT_4_ receptors, but also noted that it took more time them to reach a plateau than 5-HT in 5-HT_4_-TG as we had before noted for cisapride or prucalopride (Keller et al. 2018).

Moreover, we demonstrated that bufotenin raised the phosphorylation state of phospholamban. We had previously reported that 5-HT increased the phosphorylation state of phospholamban in isolated atrial preparation and perfused hearts of 5-HT_4_-TG (Gergs et al. 2010) but also in isolated human atrial preparations (Gergs et al. 2009). This is mechanistically relevant because increased phosphorylation of phospholamban (Tada et al. 1976) can explain, at least in part, why bufotenin reduced time to relaxation and increased rate of tension relaxation in atrial and ventricular preparations from 5-HT_4_-TG mice: phosphorylated phospholamban increases the rate at which calcium cations are pumped from the cytosol into the sarcoplasmic reticulum, less calcium cations bind to the myofilaments and myofilaments relax faster (Hamstra et al. 2020; Tada et al. 1976). In this context, one could ask if the phosphorylation status of contractile proteins like troponin inhibitor (TnI) are also increased. However, here we chose to study the phosphorylation of phospholamban instead of TnI because basal unstimulated PLB phosphorylation is very low using antibodies. In contrast, basal phosphorylation of TnI is quite high under basal conditions (Gergs et al. 2019b). Hence, with an agonist, it is technically much easier to measure an increase of PLB phosphorylation. This could be seen as a drawback of the study, but on the other hand, PLB and TnI phosphorylation measure the same biochemical pathway (Fig. 1). They mirror the activity of PKA. Furthermore, it can be asked why we noted contractile effects of tryptamine even in the absence of pargyline in contrast to Medhurst and Kaumann (1993) who failed to measure any effect of tryptamine in the absence of pargyline (Medhurst and Kaumann 1993). The following explanation seems plausible. Pargyline at 50 µM has been reported to inhibit irreversibly both MAO A and MAO B in vitro with IC50-values (half inhibitory concentrations) of about 0.012 µM and 0.0082 µM respectively (Fisar et al. 2010). Hence, 50 µM pargyline will inhibit both isoenzymes of MAO. Tryptamine is metabolized and inactivated by both MAO A and MAO B in vitro (Kalgutkar et al. 2001). However, MAO A is much less active compared to MAO B in mouse heart than in other mammalian species (Villeneuve et al. 2013). Moreover, total MAO activity in mouse heart is low compared to pig heart (Boomsma et al. 2000; Villeneuve et al. 2013). Hence, less tryptamine is probably degraded in the mouse heart than in the pig heart and therefore we did measure inotropic and chronotropic effects of tryptamine given alone. However, if we added pargyline, tryptamine (and bufotenin) were more potent, because apparently, though MAO activity is low in mouse heart, it is sufficient to inactivate at least some tryptamine and bufotenin molecules. At least in rat, it has been noted that bufotenin injected in rats, increased tissue levels of not only bufotenin in, e.g., heart and lung and brain, but also that in these tissues the putative metabolite 5-hydroxy-indole-acetic acid was formed (Fuller et al. 1995). The level of the metabolite was lower when rat were pre-treated with pargyline or other MAO inhibitors (Fuller et al. 1995). This suggests that bufotenin metabolism at least in rat heart occurs and we extend these findings to mouse atrium. We also showed that the inotropic effects of bufotenin could be antagonized by tropisetron, a compound known also to block 5-HT_4_ receptors. Hence, we can claim that the effects of bufotenin are 5-HT_4_ mediated by using two lines of argument: firstly, they occurred only in 5-HT_4_-TG and not WT and secondly they are blocked by 5-HT_4_ antagonist.

Medhurst and Kaumann (1993) showed that bufotenin and tryptamine elevated the beating rate in isolated right atrial preparations of pigs via 5-HT_4_ receptors (Medhurst and Kaumann 1993). We confirm in our model their findings and extend them to inotropic effects in cardiac preparations from transgenic mice and the human heart. Like in our study, in their study, bufotenin (pD2 = 5.95) was more potent than tryptamine (pD2 = 4.87) (Medhurst and Kaumann 1993). Thus, like them, we also noted that tryptamine was less potent than bufotenin to increase the beating rate in right atrial preparations from 5-HT_4_-TG, indicating that we used the proper model to study cardiac effects of bufotenin (Table 2). A new finding here is that tryptamine was also less potent than bufotenin to increase force of contraction, at least in left atrial preparations from 5-HT_4_-TG.

## Clinical relevance

To the best of our knowledge, we show for the first time that bufotenin can increase force of contraction in isolated human atrium. This cardiac effect might play a clinical role. Bufotenin can be taken orally to induce hallucinogenic effects but perorally high doses must be given in human because bufotenin seems to undergo a strong first pass effect (probably via MAO activity in the gastrointestinal tract), as much higher per-oral doses (100 mg) than parenteral doses (10 mg) are needed in humans to bring about hallucinogenic effects (Ott 2001). Bufotenin has been tested in animals against rabies (Vigerelli et al. 2014) which might lead to applications of bufotenin in humans. Plasma levels of bufotenin were elevated in patients with autism and schizophrenia (Emanuele et al. 2010) and from our data one might hypothesize that these high levels of bufotenin might lead to tachycardia in untreated patients. Hence, it might be worthwhile to help some of these patients with 5-HT_4_ receptor antagonists. Bufotenin has some beneficial effects in depressive patients (Uthaug et al. 2019). However, there is currently no accepted clinical indication for bufotenin. However, bufotenin itself and frog skins or plants containing bufotenin are sometimes used as “recreational drugs” and have led to intoxications over decades (Chamakura 1994; Davis et al. 2018; Shen et al. 2010).

Bufotenin is, moreover, indirectly clinically relevant because it is an important active metabolite of the hallucinogenic compound 5-methoxy N,N-dimethyltryptamine (found in plants) and thus might be formed by metabolism in subjects using the prodrug (Shen et al. 2010) (Fig. 1). Our data might argue that intoxications with bufotenin or its precursor 5-methoxy N,N-dimethyltryptamine can involve cardiac side effects that could be treated by 5-HT_4_ receptor antagonists. One could treat bufotenin-intoxicated patients with tropisetron: tropisetron is typically regarded as a 5-HT_3_ receptor antagonist but tropisetron blocks also human 5-HT_4_ receptors (Kaumann et al. 1990) and tropisetron is approved for usage in humans in many countries. Alternatively, one can use the specific 5-HT_4_ receptor antagonist piboserod (Kjekshus et al. 2009), that has been used at least in one heart failure study in humans and thus might be used off-label should the need arise in the patient.

In summary, using the 5-HT_4_-TG model, we detected cardiac inotropic and chronotropic effects for a hallucinogenic drug that is not intended to act on the heart, namely bufotenin.

## Data Availability

The data of this study are available from the corresponding author upon reasonable request.

## References

[CR1] Almaula N, Ebersole BJ, Ballesteros JA, Weinstein H, Sealfon SC (1996). Contribution of a helix 5 locus to selectivity of hallucinogenic and nonhallucinogenic ligands for the human 5-hydroxytryptamine2A and 5-hydroxytryptamine2C receptors: direct and indirect effects on ligand affinity mediated by the same locus. Mol Pharmacol.

[CR2] Boknik P, Drzewiecki K, Eskandar J, Gergs U, Hofmann B, Treede H, Grote-Wessels S, Fabritz L, Kirchhof P, Fortmüller L, Müller FU, Schmitz W, Zimmermann N, Kirchhefer U, Neumann J (2019). Evidence for arrhythmogenic effects of A2A-Adenosine receptors. Front Pharmacol.

[CR3] Boomsma F, van Dijk J, Bhaggoe UM, Bouhuizen AM, van den Meiracker AH (2000). Variation in semicarbazide-sensitive amine oxidase activity in plasma and tissues of mammals. Comp Biochem Physiol C Pharmacol Toxicol Endocrinol.

[CR4] Chamakura RP (1994). Bufotenine - a hallucinogen in ancient snuff powders of South America and a drug of abuse on the streets of New York City. Forensic Sci Rev.

[CR5] Chilton WS, Bigwood J, Jensen RE (1979). Psilocin, bufotenine and serotonin: historical and biosynthetic observations. J Psychedelic Drugs.

[CR6] Davis AK, Barsuglia JP, Lancelotta R, Grant RM, Renn E (2018). The epidemiology of 5-methoxy- N, N-dimethyltryptamine (5-MeO-DMT) use: benefits, consequences, patterns of use, subjective effects, and reasons for consumption. J Psychopharmacol.

[CR7] Dumuis A, Sebben M, Bockaert J (1988). Pharmacology of 5-hydroxytryptamine-1A receptors which inhibit cAMP production in hippocampal and cortical neurons in primary culture. Mol Pharmacol.

[CR8] Emanuele E, Colombo R, Martinelli V, Brondino N, Marini M, Boso M, Barale F, Politi P (2010). Elevated urine levels of bufotenine in patients with autistic spectrum disorders and schizophrenia. Neuro Endocrinol Lett.

[CR9] Faust ES (1902). Ueber Bufonin und Bufotalin, die wirksamen Bestandtheile des Krötenhautdrüsensecretes. Arc Exp Pathol Pharm.

[CR10] Fisar Z, Hroudová J, Raboch J (2010). Inhibition of monoamine oxidase activity by antidepressants and mood stabilizers. Neuro Endocrinol Lett.

[CR11] Forsström T, Tuominen J, Karkkäinen J (2001). Determination of potentially hallucinogenic N-dimethylated indoleamines in human urine by HPLC/ESI-MS-MS. Scand J Clin Lab Invest.

[CR12] Fuller RW, Snoddy HD, Perry KW (1995). Tissue distribution, metabolism and effects of bufotenine administered to rats. Neuropharmacology.

[CR13] Gergs U, Neumann J, Simm A, Silber R-E, Remmers FO, Läer S (2009). Phosphorylation of phospholamban and troponin I through 5-HT4 receptors in the isolated human atrium. Naunyn Schmiedebergs Arch Pharmacol.

[CR14] Gergs U, Baumann M, Böckler A, Buchwalow IB, Ebelt H, Fabritz L, Hauptmann S, Keller N, Kirchhof P, Klöckner U, Pönicke K, Rueckschloss U, Schmitz W, Werner F, Neumann J (2010). Cardiac overexpression of the human 5-HT4 receptor in mice. Am J Physiol Heart Circ Physiol.

[CR15] Gergs U, Böckler A, Ebelt H, Hauptmann S, Keller N, Otto V, Pönicke K, Schmitz W, Neumann J (2013). Human 5-HT_4_receptor stimulation in atria of transgenic mice. Naunyn Schmiedebergs Arch Pharmacol.

[CR16] Gergs U, Fritsche J, Fabian S, Christ J, Neumann J (2017). Desensitization of the human 5-HT4 receptor in isolated atria of transgenic mice. Naunyn Schmiedebergs Arch Pharmacol.

[CR17] Gergs U, Jung F, Buchwalow IB, Hofmann B, Simm A, Treede H, Neumann J (2017). Pharmacological and physiological assessment of serotonin formation and degradation in isolated preparations from mouse and human hearts. Am J Physiol Heart Circ Physiol.

[CR18] Gergs U, Rothkirch D, Hofmann B, Treede H, Robaye B, Simm A, Müller CE, Neumann J (2018). Mechanism underlying the contractile activity of UTP in the mammalian heart. Eur J Pharmacol.

[CR19] Gergs U, Mangold W, Langguth F, Hatzfeld M, Hauptmann S, Bushnaq H, Simm A, Silber R-E, Neumann J (2019). Alterations of protein expression of phospholamban, ZASP and plakoglobin in human atria in subgroups of seniors. Sci Rep.

[CR20] Gergs U, Bernhardt G, Buchwalow IB, Edler H, Fröba J, Keller M, Kirchhefer U, Köhler F, Mißlinger N, Wache H, Neumann J (2019). Initial characterization of transgenic mice overexpressing human histamine H2 receptors. J Pharmacol Exp Ther.

[CR21] Gergs U, Jahn T, Werner F, Köhler C, Köpp F, Großmann C, Neumann J (2019c) Overexpression of protein phosphatase 5 in the mouse heart: reduced contractility but increased stress tolerance - Two sides of the same coin? PLoS ONE 14:e0221289. 10.1371/journal.pone.0221289 10.1371/journal.pone.0221289 PMC6699691 31425567

[CR22] Gergs U, Gerigk T, Wittschier J, Schmidbaur CT, Röttger C, Mahnkopf M, Edler H, Wache H, Neumann J (2021a) Influence of serotonin 5-HT4 receptors on responses to cardiac stressors in transgenic mouse models. Biomedicines 9. 10.3390/biomedicines9050569 10.3390/biomedicines9050569 PMC8158346 34070090

[CR23] Gergs U, Brückner T, Hofmann B, Neumann J (2021b) The proarrhythmic effects of hypothermia in atria isolated from 5-HT4-receptor-overexpressing mice. Eur J Pharmacol 906:174206. 10.1016/j.ejphar.2021b.174206 10.1016/j.ejphar.2021.174206 34048737

[CR24] Hamstra SI, Whitley KC, Baranowski RW, Kurgan N, Braun JL, Messner HN, Fajardo VA (2020). The role of phospholamban and GSK3 in regulating rodent cardiac SERCA function. Am J Physiol Cell Physiol.

[CR25] Handovsky H (1920). Ein Alkaloid im Gifte von Bufo vulgaris. Arch Exp Pathol Phar.

[CR26] Hoshino T, Shimodaira K (1935) Synthese des Bufotenins und über 3-Methyl-3-β-oxyäthyl-indolenin. Synthesen in der Indol-Gruppe. XIV. Liebigs Ann Chem 520:19–30. 10.1002/jlac.19355200104

[CR27] Kalgutkar AS, Dalvie DK, Castagnoli N, Taylor TJ (2001). Interactions of nitrogen-containing xenobiotics with monoamine oxidase (MAO) isozymes A and B: SAR studies on MAO substrates and inhibitors. Chem Res Toxicol.

[CR28] Kärkkäinen J, Forsström T, Tornaeus J, Wähälä K, Kiuru P, Honkanen A, Stenman UH, Turpeinen U, Hesso A (2005). Potentially hallucinogenic 5-hydroxytryptamine receptor ligands bufotenine and dimethyltryptamine in blood and tissues. Scand J Clin Lab Invest.

[CR29] Kaumann AJ (1990). Piglet sinoatrial 5-HT receptors resemble human atrial 5-HT4-like receptors. Naunyn-Schmiedeberg's Arch Pharmacol.

[CR30] Kaumann AJ, Levy FO (2006). 5-hydroxytryptamine receptors in the human cardiovascular system. Pharmacol Ther.

[CR31] Kaumann AJ, Sanders L, Brown AM, Murray KJ, Brown MJ (1990). A 5-hydroxytryptamine receptor in human atrium. Br J Pharmacol.

[CR32] Keller N, Dhein S, Neumann J, Gergs U (2018). Cardiovascular effects of cisapride and prucalopride on human 5-HT4 receptors in transgenic mice. Arch Exp Pathol Pharm.

[CR33] Kirchhefer U, Baba HA, Hanske G, Jones LR, Kirchhof P, Schmitz W, Neumann J (2004). Age-dependent biochemical and contractile properties in atrium of transgenic mice overexpressing junctin. Am J Physiol Heart Circ Physiol.

[CR34] Kjekshus JK, Torp-Pedersen C, Gullestad L, Køber L, Edvardsen T, Olsen IC, Sjaastad I, Qvigstad E, Skomedal T, Osnes J-B, Levy FO (2009). Effect of piboserod, a 5-HT4 serotonin receptor antagonist, on left ventricular function in patients with symptomatic heart failure. Eur J Heart Fail.

[CR35] Lewin L (1924) Phantastica : die betäubenden und erregenden Genussmittel ; für Ärzte und Nichtärzte / von L. Lewin. Stilke

[CR36] Medhurst AD, Kaumann AJ (1993). Characterization of the 5-HT4 receptor mediating tachycardia in piglet isolated right atrium. Br J Pharmacol.

[CR37] Miller MJ, Albarracin-Jordan J, Moore C, Capriles JM (2019). Chemical evidence for the use of multiple psychotropic plants in a 1,000-year-old ritual bundle from South America. Proc Natl Acad Sci U S A.

[CR38] Moretti C, Gaillard Y, Grenand P, Bévalot F, Prévosto J-M (2006). Identification of 5-hydroxy-tryptamine (bufotenine) in takini (Brosimumacutifolium Huber subsp. acutifolium C.C. Berg, Moraceae), a shamanic potion used in the Guiana Plateau. J Ethnopharmacol.

[CR39] Neumann J, Boknik P, DePaoli-Roach AA, Field LJ, Rockman HA, Kobayashi YM, Kelley JS, Jones LR (1998). Targeted overexpression of phospholamban to mouse atrium depresses Ca2+ transport and contractility. J Mol Cell Cardiol.

[CR40] Neumann J, Boknik P, Matherne GP, Lankford A, Schmitz W (2003). Pertussis toxin sensitive and insensitive effects of adenosine and carbachol in murine atria overexpressing A(1)-adenosine receptors. Br J Pharmacol.

[CR41] Neumann J, Käufler B, Gergs U (2019) Which phosphodiesterase can decrease cardiac effects of 5-HT4 receptor activation in transgenic mice? Naunyn-Schmiedeberg’s Arch Pharmacol 392:991–1004. 10.1007/s00210-019-01653-y 10.1007/s00210-019-01653-y 31016326

[CR42] Neumann J, Schwarzer D, Fehse C, Schwarz R, Marusakova M, Kirchhefer U, Hofmann B, Gergs U (2021). Functional interaction of H2-receptors and 5HT4-receptors in atrial tissues isolated from double transgenic mice and from human patients. Naunyn-Schmiedebergs Arch Pharmacol.

[CR43] Neumann J, Hofmann B, Gergs U (2017) Production and function of serotonin in cardiac cells. In: Serotonin - A Chemical Messenger Between All Types of Living Cells. InTech, pp 271–305

[CR44] Neumann J, Seidler T, Fehse C, Marušáková M, Hofmann B, Gergs U (2021a) Cardiovascular effects of metoclopramide and domperidone on human 5-HT4-serotonin-receptors in transgenic mice and in human atrial preparations. Eur J Pharmacol 901:174074. 10.1016/j.ejphar.2021a.174074 10.1016/j.ejphar.2021.174074 33811834

[CR45] Ott J (2001). Pharmañopo-psychonautics: human intranasal, sublingual, intrarectal, pulmonary and oral pharmacology of bufotenine. J Psychoactive Drugs.

[CR46] Phisalix C, Bertrand G (1893) Toxicité comparée du sang et du venin de crapaud commun, considérée au point de vue de la sécrétion interne des glandes cutanées de cet animal. C R Soc Biol 45:477–479

[CR47] Boknik P, Drzewiecki K, Eskandar J, Gergs U, Grote-Wessels S, Fabritz L, Kirchhof P, Müller FU, Stümpel F, Schmitz W, Zimmermann N, Kirchhefer U, Neumann J (2018). Phenotyping of mice with heart specific overexpression of A2A-Adenosine receptors: evidence for cardioprotective effects of A2A-Adenosine receptors. Front Pharmacol.

[CR48] Shen H-W, Jiang X-L, Winter JC, Yu A-M (2010). Psychedelic 5-methoxy-N, N-dimethyltryptamine: metabolism, pharmacokinetics, drug interactions, and pharmacological actions. Curr Drug Metab.

[CR49] Siegel DM, McDaniel SH (1991). The frog prince: tale and toxicology. Am J Orthopsychiatry.

[CR50] Suzuki O, Katsumata Y, Oya M (1981). Characterization of eight biogenic indoleamines as substrates for type A and type B monoamine oxidase. Biochem Pharmacol.

[CR51] Tada M, Kirchberger MA, Katz AM (1976). Regulation of calcium transport in cardiac sarcoplasmic reticulum by cyclic AMP-dependent protein kinase. Recent Adv Stud Cardiac Struct Metab.

[CR52] Titeler M, Lyon RA, Glennon RA (1988). Radioligand binding evidence implicates the brain 5-HT2 receptor as a site of action for LSD and phenylisopropylamine hallucinogens. Psychopharmacology.

[CR53] Uthaug MV, Lancelotta R, van Oorsouw K, Kuypers KPC, Mason N, Rak J, Šuláková A, Jurok R, Maryška M, Kuchař M, Páleníček T, Riba J, Ramaekers JG (2019). A single inhalation of vapor from dried toad secretion containing 5-methoxy-N, N-dimethyltryptamine (5-MeO-DMT) in a naturalistic setting is related to sustained enhancement of satisfaction with life, mindfulness-related capacities, and a decrement of psychopathological symptoms. Psychopharmacology.

[CR54] Vigerelli H, Sciani JM, Jared C, Antoniazzi MM, Caporale GMM, Da Silva AdCR, Pimenta DC (2014). Bufotenine is able to block rabies virus infection in BHK-21 cells. J Venom Anim Toxins Incl Trop Dis.

[CR55] Villalón CM, den Boer MO, Heiligers JP, Saxena PR (1990). Mediation of 5-hydroxytryptamine-induced tachycardia in the pig by the putative 5-HT4 receptor. Br J Pharmacol.

[CR56] Villeneuve C, Guilbeau-Frugier C, Sicard P, Lairez O, Ordener C, Duparc T, de Paulis D, Couderc B, Spreux-Varoquaux O, Tortosa F, Garnier A, Knauf C, Valet P, Borchi E, Nediani C, Gharib A, Ovize M, Delisle M-B, Parini A, Mialet-Perez J (2013). p53-PGC-1α pathway mediates oxidative mitochondrial damage and cardiomyocyte necrosis induced by monoamine oxidase-a upregulation: role in chronic left ventricular dysfunction in mice. Antioxid Redox Signal.

[CR57] Wieland H, Konz W, Mittasch H (1934) Die Konstitution von Bufotenin und Bufotenidin. Über Kröten-Giftstoffe. VII. Justus Liebigs Ann Chem 513:1–25. 10.1002/jlac.19345130102

